# EFFECTS OF MARITAL STATUS AND ETHNICITY ON NEUROCOGNITIVE FUNCTIONING IN A RURAL OLDER POPULATION

**DOI:** 10.1093/geroni/igad104.2943

**Published:** 2023-12-21

**Authors:** Lauren Elliott, Jonathan Singer, Peter Rerick, Carol Fadalla, Elisabeth McLean, Alayna Jump, Veronica Molinar-Lopez, Volker Neugebauer

**Affiliations:** Texas Tech University, Lubbock, Texas, United States; Texas Tech University, Lubbock, Texas, United States; Oklahoma City University, Oklahoma City, Oklahoma, United States; Texas Tech University, Lubbock, Texas, United States; Texas Tech University, Lubbock, Texas, United States; Texas Tech University, Lubbock, Texas, United States; Texas Tech University Health Sciences Center, Lubbock, Texas, United States; Garrison Institute on Aging, Lubbock, Texas, United States

## Abstract

Research indicates being married is related to better physical and psychological health. Yet, little is known regarding the relationship between marital status and neurocognitive functioning and whether it differs based on ethnicity (Hispanic vs. non-Hispanic). Data from 1864 participants (Mage=59.68, SD=12.21), who were mostly Hispanic (n=1053), women (n=1295), and married (n=1,125) from Project FRONTIER (Facing Rural Obstacles to Healthcare Now Through Intervention, Education, & Research) were analyzed. Neuropsychological testing comprised RBANS, Trails Making Test, and Clock Drawing. Participants were dichotomized into married and unmarried (i.e., widowed, divorced, separated, never married). There was a significant interaction between Hispanic identity and marital status on overall neurocognitive functioning (F(1,1480) =4.79, p < .05, ηp2=.003). For non-Hispanic individuals, married individuals had higher overall neurocognitive functioning compared to the unmarried individuals, whereas neurocognitive functioning for Hispanic individuals did not significantly differ between married and unmarried individuals. There were significant main effects as married individuals (M=84.95, SD=15.56) had greater overall neurocognitive functioning than unmarried individuals (M=83.47, SD=15.86; F(1,1480) = 14.67, p < .001, ηp2=.01), and Hispanic individuals (M=78.02, SD=14.25) had lower overall neurocognitive functioning than non-Hispanic individuals (M=91.43, SD=15.07; F(1,1480) = 284.99, p < .001, ηp2=.16). Hispanics living in rural areas experience additional stressors that could lead to worse neurocognitive functioning, which is supported by the Lifespan Biopsychosocial Model of Cumulative Vulnerability and Minority Health, which postulates that race/ethnicity/SES-related stressors exacerbate the impact of other life stressors. Reduction of stress on rural Hispanics should be a priority as it could positively affect their neurocognitive functioning.

